# The efficacy and safety of Ivermectin in patients with mild and moderate COVID-19: A structured summary of a study protocol for a randomized controlled trial

**DOI:** 10.1186/s13063-020-04988-7

**Published:** 2021-01-04

**Authors:** Fatemeh Sadat Hosseini, Alireza Malektojari, Sara Ghazizadeh, Mehdi Hassaniazad, Parivash Davoodian, Habib Dadvand, Amin Reza Nikpoor, Sara Nikoofal-Sahlabadi, Sara Kahoori, Mojtaba Sepandi, Soheil Hassanipour, Mohammad Fathalipour

**Affiliations:** 1 Student Research Committee, Hormozgan University of Medical Sciences, Bandar Abbas, Iran; 2 Infectious and Tropical Diseases Research Center, Hormozgan Health Institute, Hormozgan University of Medical Sciences, Bandar Abbas, Iran; 3 Molecular Medicine Research Center, Hormozgan Health Institute, Hormozgan University of Medical Sciences, Bandar Abbas, Iran; 4 Department of Pharmaceutics, Faculty of Pharmacy, Hormozgan University of Medical Sciences, Bandar Abbas, Iran; 5 Department of Pharmacology and Toxicology, Faculty of Pharmacy, Hormozgan University of Medical Sciences, Bandar Abbas, Iran; 6 Department of Epidemiology and Biostatistics, Faculty of Health, Baqiyatallah University of Medical Sciences, Tehran, Iran; 7 Gastrointestinal and Liver Diseases Research Center, Guilan University of Medical Sciences, Rasht, Iran

**Keywords:** COVID-19, Randomized controlled trial, Protocol, Ivermectin, Hospitalization Mechanical ventilation, Clinical symptoms

## Abstract

**Objectives:**

We will evaluate the efficacy and safety of Ivermectin in patients with mild and moderately severe COVID-19.

**Trial design:**

This is a phase 3, single-center, randomized, open-label, controlled trial with a 2-arm parallel-group design (1:1 ratio).

**Participants:**

The Severe Acute Respiratory Syndrome Departments of the Shahid Mohammadi Hospital, Bandar Abbas, Iran, will screen for patients age ≥ 20 years and weight ≥35 kg for the following criteria:

*Inclusion criteria for patients with mild COVID-19 symptoms (outpatients)*

1. Diagnosed mild pneumonia using computed tomography (CT) and/or chest X-ray (CX-R) imaging, not requiring hospitalization.
2. Signing informed consent.

*Inclusion criteria for patients with moderate COVID-19 symptoms (inpatients)*

1. Confirmed infection using PCR.
2. Diagnosed moderate pneumonia using CT and/or CXR imaging, requiring hospitalization.
3. Hospitalized ≤ 48 hours.
4. Signing informed consent.

Exclusion criteria

1. Severe and critical pneumonia due to COVID-19.
2. Underlying diseases, including AIDS, asthma, loiasis, and severe liver and kidney disease.
3. Use of anticoagulants (e.g., warfarin) and ACE inhibitors (e.g., captopril).
4. History of drug allergy to Ivermectin.
5. Pregnancy or breastfeeding.

**Intervention and comparator:**

Intervention groups: Outpatient and inpatient groups will receive the standard treatment regimen for mild and moderate COVID-19, based on the Iranian Ministry of Health and Medical Education's protocol, along with oral Ivermectin (MSD Company, France) at a single dose of 0.2 mg/kg.

Control groups: The outpatient group will receive hydroxychloroquine sulfate (Amin Pharmaceutical Company, Iran) at a dose of 400 mg twice a day for the first day and 200 mg twice a day for seven subsequent days. The inpatient group will receive 200/50 mg Lopinavir/Ritonavir (Heterd Company, India) twice a day for the seven days, plus five doses of 44 mcg Interferon beta-1a (CinnaGen, Iran) every other day.

Other supportive and routine care will be the same in both outpatient and inpatient groups.

**Main outcome:**

The primary outcomes are composite and include the improvement of clinical symptoms and need for hospitalization for outpatient groups, and the length of hospital stay until discharge, the need for ICU admission until discharge, and the need for mechanical ventilation for inpatient groups within seven days of randomization.

The secondary outcome is the incidence of serious adverse drug reactions within seven days of randomization.

**Randomization:**

Patients in both outpatient (mild) and inpatient (moderate) groups will be randomized into the treatment and control groups based on the following method. A simple randomization method and table of random numbers will be used. If the selected number is even, the patient is allocated to the treatment group, and if it is odd, the patient is allocated to the control group in a 1:1 ratio.

**Blinding (masking):**

This is an open-label study, and there is not blinding.

Numbers to be randomized (sample size)

A total number of 120 patients (60 outpatients and 60 patients) will be randomized into two groups (30 patients in each of the intervention groups and 30 patients in each of the control groups).

**Trial Status:**

The protocol is Version 1.0, November 17, 2020. Recruitment began November 25, 2020, and is anticipated to be completed by February 25, 2021.

**Trial registration:**

This clinical trial has been registered in the Iranian Registry of Clinical Trials (IRCT). The registration number is “IRCT20200506047323N6”. The registration date is November 17, 2020.

**Full protocol:**

The full protocol is attached as an additional file, accessible from the Trials website (Additional file 1). In the interest in expediting the dissemination of this material, the familiar formatting has been eliminated; this letter serves as a summary of the key elements of the full protocol.

**Supplementary Information:**

The online version contains supplementary material available at 10.1186/s13063-020-04988-7.

## Supplementary Information

**Additional file 1.** Full Study Protocol.

## Data Availability

The corresponding author has access to the final trial information, and the data will be available on reasonable request (Contact: M.fathalipour@hums.ac.ir).

