# Supplementary material for: The efficacy and safety of Ivermectin in patients with mild and moderate COVID-19: A structured summary of a study protocol for a randomized controlled trial
Source: Trials. 2021 Jan 4;22:4. doi: 10.1186/s13063-020-04988-7 (PMC7780209; doi:10.1186/s13063-020-04988-7)
Supplement: Supplementary file 1 — Additional file 1. Full Study Protocol. [file 13063_2020_4988_MOESM1_ESM.pdf]

Protocol

This trial protocol has been provided by the authors to give readers additional information about their work.

The efficacy and safety of Ivermectin in patients with COVID-19: A structured summary of a study protocol for a randomized controlled trial

Fatemeh Sadat Hosseini [1], Alireza Malektojari [2], Sara Ghazizadeh [2], Mehdi Hassaniazad [2], Parivash Davoodian [2], Habib Dadvand [2], Amin Reza Nikpoor [3], Sara Nikoofal-Sahlabadi [4], Soheil Hassanipour [5], Sara Kahoori [6], Mohammad Fathalipour [6,7]

[1] *Student Research Committee, Hormozgan University of Medical Sciences, Bandar Abbas, Iran.*

[2] *Infectious and Tropical Diseases Research Center, Hormozgan Health Institute, Hormozgan University of Medical Sciences, Bandar Abbas, Iran.*

[3] *Molecular Medicine Research Center, Hormozgan Health Institute, Hormozgan University of Medical Sciences, Bandar Abbas, Iran.*

[4] *Department of Pharmaceutics, Faculty of Pharmacy, Hormozgan University of Medical Sciences, Bandar Abbas, Iran.*

[5] *Gastrointestinal and Liver Diseases Research Center, Guilan University of Medical Sciences, Rasht, Iran.*

[6] *Department of Pharmacology and Toxicology, Faculty of Pharmacy, Hormozgan University of Medical Sciences, Bandar Abbas, Iran.*

[7] *Endocrinology and Metabolic Research Center, Hormozgan University of Medical Sciences, Bandar Abbas, Iran.*

بررسی اثربخشی و ایمنی ایورمکتین در بیماران مبتلا به COVID-19: یک کارآزمایی بالینی تصادفی

شناسنامه طرح

عنوان طرح - فارسی	بررسی اثربخشی و ایمنی ایورمکتین در بیماران مبتلا به COVID-19: یک کارآزمایی بالینی تصادفی
Title	The efficacy and safety of Ivermectin in patients with COVID-19: a randomized clinical trial
چکیده پروپوزال	شیوع عفونت سندرم تنفسی شدید حاد ناشی از کرونا ویروس (SARS-CoV-2) و بیماری کورونا ویروس ۲۰۱۹ (COVID-19) ناشی از آن، از دسامبر سال ۲۰۱۹ در چین گزارش شده است. بیش از ۱۶ درصد از بیماران مبتلا به این بیماری دچار سندرم شدید تنفسی می شوند، و حدود ۱-۲ درصد از این بیماران دچار مرگ و میر می شوند. تاکنون هیچ درمانی اختصاصی گزارش نشده است. از دارو های متعددی در معالجه این بیماری استفاده شده است، اما اثربخشی این داروها همچنان بحث برانگیز است. مطالعه حاضر با هدف بررسی اثربخشی و ایمنی لوپیناویر/ریتوناووير در مقایسه با لوپیناویر/ریتوناووير و ایورمکتین در بیماران مبتلا به کووید-۱۹ انجام خواهد شد. این کارآزمایی بالینی آینده نگر و تصادفی با برچسب باز روی بیماران مبتلا به کووید-۱۹ انجام خواهد شد. بیماران به طور تصادفی به نسبت ۱:۱ در گروه های لوپیناویر/ریتوناویر (لوپیناویر ۲۰۰ میلی گرم/ریتوناویر ۵۰ میلی گرم دو بار در روز برای ۷ روز) و گروه لوپیناویر/ریتوناویر و ایورمکتین (به صورت تک دوز mg/kg 2/0) تقسیم خواهند شد. پس از اتمام دوره مطالعه پیامد هایی همچون علائم بیماری طول مدت بستری و نیاز به ونتیلاسیون مورد بررسی قرار خواهند گر
abstract	An outbreak of severe acute respiratory syndrome coronavirus 2 (SARS-CoV-2) infection and its caused coronavirus disease 2019 (COVID-19) has been reported in China since December 2019. More than 16% of patients developed acute respiratory distress syndrome, and the fatality ratio was about 1-2%. No specific treatment has been reported. Several drugs has been evaluated in treatment of COVID However, the efficacy of these medications remains controversial. The present study aims to assess the e the efficacy and safety of Lopinavir/Ritonavir and Ivermectin in patients with covid-19. A perspective randomized open-label clinical trial will be conducted on 60 hospitalized adult patients with covid-19. Patients will be randomly assigned in a 1:1 ratio to receive, whether Lopinavir/Ritonavir (lopinavir 200 mg and ritonavir 50 mg twice a day twice a day for seven days), or Lopinavir/Ritonavir plus Ivermectin (single dose of 2.0 mg/kg(The main outcomes will be improvement of clinical symptoms duration of hospitalization
دانشکده/مرکز	پژوهشکده سلامت – مرکز تحقیقات بیماریهای عفونی و گرمسیری
تحقیق در نظام سلامت	☑
آیا طرح برگرفته از پایان‌نامه می باشد؟	☐
مقطع پایان نامه	
نوع پایان نامه	کارآزمایی بالینی و مداخله ای
مدت اجرا - ماه	۶

مدت اجراء - روز	۱۸۰
نوع پژوهش	طرح تحقیقاتی
نوع طرح	☑ کاربردی
نوع مطالعه	☑ کارآزمایی بالینی - Clinical Trial
نحوه پرداخت	آزمایشگاهی
کد IRCT	IRCT20200506047323N6
قرارداد پژوهشی مشترک	☐

دانشکده/مرکز	نوع ارتباط با مرکز	نحوه همکاری
پژوهشکده سلامت(عفونی و گرمسیری-پزشکی مولکولی-ارتقاء سلامت)	وارد کننده	

متون پیشنهاد

عنوان	متن
بیان مساله	بیماری کووید ۱۹ (COVID-19) که به آن بیماری تنفسی حاد ناشی از کرونا ویروس (SARS-CoV-2) نیز گفته می‌شود، بیماری عفونی است که در دسامبر سال ۲۰۱۹، در ووهان چین پدیدار گردید و به سرعت در چین و خارج از آن شیوع یافت (۱, ۲). به دنبال شیوع این بیماری در سایر نقاط جهان، سازمان بهداشت جهانی در مارس سال ۲۰۲۰ اپیدمی COVID-19 را به عنوان پاندمی اعلام کرد (۳).<br><br>شایع ترین علائم این بیماری شامل تب، سرفه و تنگی نفس می باشد (۴). با این حال، علائم دیگری همچون خستگی مفرط، درد عضلانی، سردرد، ضعف و بی حالی، کاهش حس بویایی و چشایی، اسهال، اسپاسم شکمی، تهوع و استفراغ ممکن است در بیماران بروز پیدا کند (۴-۶). علائم به طور معمول ۵ روز پس از تماس با ویروس شروع می شوند (۷). در حالی که اکثر بیماران دچار علائم خفیف می شوند، در برخی موارد این بیماری به پنمونی ویروسی و نارسایی چند اندامی منجر می شود (۸). طبق مطالعاتی که اخیرا در چین انجام شده است، حدود ۸۰ درصد از بیماران مبتلا، بیماری خفیف را تجربه می کنند و میزان مرگ و میر حدود ۲/۳ درصد است. اما در بیماران ۷۰ تا ۷۹ ساله این میزان به ۱۴/۸ درصد و در افراد بالای ۸۰ سال به ۱۸/۰ درصد افزایش می

متن	عنوان
یابد (۹). در ۲ ژوئیه ۲۰۲۰، حدود ۱۰/۶ میلیون مورد ابتلا به این بیماری در بیش از ۱۸۸ کشور گزارش شده است، که مرگ بیش از ۵۱۶۰۰۰ نفر را به همراه داشته است. البته از مبتلایان به این بیماری بیش از ۵/۶ میلیون نفر بهبودی کامل بدست آورده اند.<br><br>بر این اساس، تعداد چشم گیری از ناقلان بدون علامت در جمعیت وجود دارد، و بنابراین احتمالاً میزان مرگ و میر بیش از حد ارزیابی می شود. نهایتاً، یک درمان مؤثر برای معالجه بیماران علامت دار در جهت کاهش میزان مرگ و میر به ویژه در گروه های پرخطر، تخفیف علایم بیماری و کاهش احتمال انتقال ویروس در جامعه یک نیاز فوری قلمداد می شود. در حال حاضر تعداد زیادی کار آزمایی بالینی جهت بررسی اثرات داروهای قدیمی ضد ویروس، ضد انگل و ضد باکتری در حال انجام است، زیرا اطلاعات فراوانی در زمینه ایمنی، عوارض جانبی، بیواکی والانسی و تداخلات دارویی این دارو ها در دسترس است. علی رغم تلاش های فراوان در این زمینه تا کنون هیچ واکسن و یا رژیم دارویی در درمان این بیماری توسط سازمان های نظارتی دنیا تائید نشده است (۱۰, ۱۱).<br><br>در میان داروهای کاندید برای درمان بیماری COVID-19، استفاده از داروهای ضد کرم و انگل موثر بر سایر بیماری ها، یک استراتژی جالب است که مورد توجه محققین قرار گرفته است.<br><br>مطالعه حاضر با هدف بررسی اثربخشی و ایمنی داروهای لوپیناویر/ریتوناویر در مقایسه با ایورمکتین با لوپیناویر/ریتوناویر در بیماران مبتلا به COVID-19 بستری شده در بیمارستان شهید محمدی شهرستان بندرعباس انجام خواهد شد.	
	تعاریف عملیاتی مفاهیم اصلی
کووید ۱۹ یک بیماری عفونی ویروسی می باشد که توسط SARS-Corona virus 2 ایجاد می شود. بیماری کووید ۱۹ برای اولین بار در دسامبر ۲۰۱۹ در ووهان چین شناسایی شد و در مارس ۲۰۲۰ توسط سازمان بهداشت جهانی یک پاندمی اعلام شد.(۱)بر اساس آمار WHO این بیماری بیش از ۲۹ میلیون مبتلا را در جهان تا سپتامبر ۲۰۲۰ داشته است.بیشترین علائم این بیماری شامل تب ، سرفه ، خستگی ، تولید خلط، تنگی نفس ، درد مفاصل و عضلات ، تهوع ،استفراغ و اسهال می باشد ، از این بین شمار بسیاری از بیماران مبتلا به نومونی به اکسیژن تراپی موقت احتیاج پیدا می کنند(۲).کووید ۱۹ یک بیماری مشترک بین انسان و حیوان می باشد و احتمال مرگ و میر در آن بین ۲ تا ۵ در صد می باشد.این بیماری از طریق تماس بین افراد ، قطرک های تنفسی می تواند منتقل شود (۳).علی رغم مطالعات فراوان تا کنون برای این بیماری داروی اختصاصی یا	بررسی متون

عنوان	متن
	واکسنی تولید نشده است.از این رو از بسیاری از دارو های غیر اختصاصی برای اثرات ضد ویروسی مانند هیدروکسی کلروکین و بسیاری از دارو های علامت درمانی برای بهبود این بیماری استفاده می شود.<br><br>ایورمکتین ؛ که یک داروی ضد کرم مورد تایید سازمان غذا و دارو می باشد ؛ در محیط in vitro پس از آلوده شدن رده های سلولی Vero-hSLAM به SARS-Corona virus 2 توانست تکثیر آن ها را پس ۵۰۰۰ بار در طی دو روز کاهش دهد (۴). ایورمکتین و هیدروکسی کلروکین با ایجاد یک محیط اسیدی و مهار ورود ویروس از طریق ایمپورتین (IMPα/β1) اثرات ضد ویروسی برجای می گذارند. ایورمکتین علاوه بر جلوگیری از ورود نوکلئیک اسید ویروس ، از انتقال پروتئین های سلول میزبان نیز جلوگیری می کند (۵).مولکول های ایورمکتین می توانند با یکدیگر تشکیل یونوفور دهند.یونوفور به مولکول هایی اطلاق می شود که محلی برای شلات کردن و به دام انداختن یون ها دارند.یونوفور تشکیل شده از ایورمکتین می تواند یون های لازم برای برخی از آنزیم های کلیدی ویروس را به دام انداخته و اینگونه آن ها را مهار کند.با این مکانیسم ایورمکتین می تواند در مراحل اولیه ی بیماری ، ویروس ها را از بین ببرد (۶).<br><br>مطالعات داکینگ مولکولی :<br><br>با توجه به مشخص شدن ساختار کریستالی پروتئین های ویروس ، مطالعات in silico نیز صورت گرفت.فایل LZG.pdb۶ که نشانگر برهمکنش پروتئین اسپایک ویروس با رسپتور سلول انسانی آن یعنی ACE-2 می باشد ، به عنوان تارگت انتخاب شد.اکتیو سایت احتمالی آن با روش اینتراکشن پروتئین –پروتئین مشخص شد و به عنوان گرید باکس نهایی انتخاب شد.آمینواسید های بسیار درگیر در برهمکنش ورود به سلول شامل آمینواسید های جدول زیر هستند که از دیتابیس Sppider استخراج شده اند.<br><br>انرژی اتصال ترکیب با اکتیو سایت احتمالی حدود -۱۰,۳ کیلوکالری برمول محاسبه شد که رقم قابل توجهی است و نشانگر افینیتی بالای ترکیب به پروتئین اسپایک ویروسی است.بنابر این افینتی بالای ایورمکتین منجر به ممانعت از اتصال آن به رسپتورACE-2 شده و ورود به سلول ویروس ، مختل می گردد.ایورمکتین به طور مثال با هیستیدین ۳۴ از رشته ی A ، تیروزین ۴۷۳ از رشته ی B ،آسپارتات از رشته ی A ،که همانطور از در جدول پیداست ، از آمینواسیدهای کلیدی انتقال ویروس به سلول هستند ، اینتراکشن برقرار می کند. در نتیجه این تداخل در ورود به سلول ویروس، تا حد زیادی از عفونت زایی ویروس می کاهد.
هدف کلی	بررسی اثربخشی و ایمنی ایورمکتین در مقایسه با رژیم درمانی استاندارد در بیماران مبتلا به COVID-19 خفیف و متوسط

متن	عنوان
• بررسی اثربخشی ایورمکتین بر بهبود علائم بالینی در بیماران مبتلا به COVID-19 خفیف در مقایسه با رژیم درمانی استاندارد<br>• بررسی اثربخشی ایورمکتین بر میزان نیاز به بستری در بیمارستان در بیماران مبتلا به COVID-19 خفیف در مقایسه با رژیم درمانی استاندارد<br>• بررسی سمیت و عوارض جانبی ایورمکتین در بیماران مبتلا به COVID-19 خفیف در مقایسه با رژیم درمانی استاندارد<br>• بررسی اثربخشی ایورمکتین بر طول مدت زمان بستری در بیماران مبتلا به COVID-19 متوسط در مقایسه با رژیم درمانی استاندارد<br>• بررسی اثربخشی ایورمکتین بر میزان نیاز به بستری در بخش مراقبت های ویژه در بیماران مبتلا به COVID-19 متوسط در مقایسه با رژیم درمانی استاندارد<br>• بررسی اثربخشی ایورمکتین بر میزان نیاز به ونتیلاسیون در بیماران مبتلا به COVID-19 متوسط در مقایسه با رژیم درمانی استاندارد<br>• بررسی سمیت و عوارض جانبی ایورمکتین در بیماران مبتلا به COVID-19 متوسط در مقایسه با رژیم درمانی استاندارد	اهداف اختصاصی
در صورت مشاهده اثرات مثبت از ایورمکتین، این دارو می توانند به بهبود کارایی رژیم های درمانی موجود کمک کند و در صورت حصول پاسخ دهی بهتر نسبت به درمان استاندارد می توان در آینده این دارو به رژیم های درمانی استاندارد جهت بهبود سریع تر علائم بالینی و یافته های آزمایشگاهی بیماران اضافه شود.<br>• انتظار می رود که این مطالعه به تدوین پروتکل درمانی در بیماری COVID-19 کمک کند.<br>• انتظار می رود که این مطالعه منجر به کاهش مشکلات درمانی دربیماری COVID-19 شود.<br>• انتظار می رود که نتایج این تحقیق منجر به راه کارهایی جهت کنترل بیماری COVID-19 در کشور شود.<br>• انتظار می رود که نتایج این تحقیق از طریق کاهش مشکلات بالینی منجر به کاهش هزینه های درمانی در بیماران COVID-19 شود.	اهداف کاربردی
• تجویز ایورمکتین باعث بهبود علائم بالینی در بیماران مبتلا به COVID-19 خفیف در مقایسه با رژیم درمانی استاندارد می شود.<br>• تجویز ایورمکتین باعث کاهش میزان نیاز به بستری در بیمارستان در بیماران مبتلا به COVID-19 خفیف در مقایسه با رژیم درمانی استاندارد می شود.<br>• ایورمکتین باعث بروز عوارض جانبی در بیماران مبتلا به COVID-19 خفیف در مقایسه با رژیم درمانی استاندارد نمی شود.<br>• تجویز ایورمکتین باعث کاهش طول مدت زمان بستری در بیماران مبتلا به COVID-19 متوسط در مقایسه با رژیم درمانی استاندارد می شود.	فرضیات (برای تحقیقات تحلیلی یا تجربی/کارآزمایی بالینی)

متن	عنوان
• تجویز ایورمکتین باعث کاهش میزان نیاز به بستری در بخش مراقبت های ویژه در بیماران مبتلا به COVID-19 متوسط در مقایسه با رژیم درمانی استاندارد می شود.<br>• تجویز ایورمکتین باعث کاهش میزان نیاز به ونتیلاسیون در بیماران مبتلا به COVID-19 متوسط در مقایسه با رژیم درمانی استاندارد می شود.<br>• تجویز ایورمکتین باعث عوارض جانبی ایورمکتین در بیماران مبتلا به COVID-19 متوسط در مقایسه با رژیم درمانی استاندارد نمی شود.	
• آیا تجویز ایورمکتین باعث بهبود علائم بالینی در بیماران مبتلا به COVID-19 خفیف در مقایسه با رژیم درمانی استاندارد می شود؟<br>• آیا تجویز ایورمکتین باعث کاهش میزان نیاز به بستری در بیمارستان در بیماران مبتلا به COVID-19 خفیف در مقایسه با رژیم درمانی استاندارد می شود؟<br>• آیا ایورمکتین باعث بروز عوارض جانبی در بیماران مبتلا به COVID-19 خفیف در مقایسه با رژیم درمانی استاندارد می شود؟<br>• آیا تجویز ایورمکتین باعث کاهش طول مدت زمان بستری در بیماران مبتلا به COVID-19 متوسط در مقایسه با رژیم درمانی استاندارد می شود؟<br>• آیا تجویز ایورمکتین باعث کاهش میزان نیاز به بستری در بخش مراقبت های ویژه در بیماران مبتلا به COVID-19 متوسط در مقایسه با رژیم درمانی استاندارد می شود؟<br>• آیا تجویز ایورمکتین باعث کاهش میزان نیاز به ونتیلاسیون در بیماران مبتلا به COVID-19 متوسط در مقایسه با رژیم درمانی استاندارد می شود؟<br>• آیا تجویز ایورمکتین باعث عوارض جانبی ایورمکتین در بیماران مبتلا به COVID-19 متوسط در مقایسه با رژیم درمانی استاندارد می شود؟	سوالات(برای تحقیقات توصیفی و تشخیصی)
کارآزمایی بالینی تصادفی برچسب باز	نوع مطالعه

عنوان	متن
روش اجرای مطالعه (جمع آوری اطلاعات- ابزار و تکنیک ها)	**طراحی مطالعه و شرکت کنندگان**<br><br>این مطالعه در قالب یک کارآزمایی بالینی تصادفی با برچسب باز[۱] بر روی ۶۰ بیمار مبتلا به بیماری COVID-19 متوسط بستری شده در بخش سندرم حاد تنفسی بیمارستان شهید محمدی شهرستان بندرعباس و ۶۰ بیمار مبتلا به COVID-19 سرپایی خفیف طراحی شده است. بیماران از ۲۵ نوامبر ۲۰۲۰ به صورت آینده نگر وارد مطالعه و پیگیری خواهند شد. با توجه به نسبت ۱:۱ بین گروه های مورد مطالعه (گروه دریافت کننده ایورمکتین و گروه دریافت کننده رژیم درمانی استاندارد)، بیماران به صورت تصادفی وارد هر یک از بازو های مداخله می شوند.<br><br>در شروع مطالعه بیماران بر اساس علائم بالینی و یافته های پاراکلینیکی در گروه های خفیف، متوسط، شدید و بحرانی تقسیم بندی می شوند (جدول ۱).<br><br>معیارهای ورود بیماران به این مطالعه برای بیماران سرپایی شامل؛ (۱) سن ۲۰ سال یا بالاتر (وزن ۲۵ کیلوگرم یا بیشتر)، (۲) رضایت آگاهانه و داوطلبانه برای شرکت در مطالعه، (۳) تشخیص قطعی بیماری COVID-19 از طریق تست PCR[۲] و/یا تست های سرلوژیک، (۴) علائم اولیه بیماری با شدت متوسط که در بیمارستان بستری شده اند (کمتر از مدت زمان ۴۸ ساعت از شروع بستری)، خواهند بود.<br><br>معیارهای ورود بیماران به این مطالعه برای بیماران بستری شامل؛ (۱) سن ۲۰ سال یا بالاتر (وزن ۲۵ کیلوگرم یا بیشتر)، (۲) رضایت آگاهانه و داوطلبانه برای شرکت در مطالعه، (۳) تشخیص قطعی بیماری COVID-19 از طریق تست PCR[۲] و/یا تست های سرلوژیک، (۴)<br><br>علائم اولیه بیماری با شدت خفیف که در بیمارستان بستری نیستند، خواهند بود.<br><br>معیارهای خروج بیماران از مطالعه نیز شامل؛ (۱) هپاتیت مزمن، سیروز کبدی، بیماری های کلستاتیک کبد، التهاب کیسه صفرا و نارسایی های شدید کلیوی، (۲) آسم و ایدز، (۳) سابقه ای از بیماری Loiasis، (۴) حساسیت شناخته شده به ایورمکتین، (۵) مصرف همزمان دارو های ضد انعقاد (مانند وارفارین) و مهار کننده های آنزیم ACE (مانند کاپتوپریل)، (۶) زنان در دوران بارداری و شیردهی و (۷) مصرف همزمان سایر داروهای ضد ویروس خواهند بود.<br><br>در ابتدای کارآزمایی، مشخصات عمومی، مشخصات دموگرافیک، آنتروپومتریک و سوابق پزشکی بیماران با استفاده از پرسشنامه استاندارد طراحی شده در این زمینه جمع آوری می شود. پس از ارائه توضیحات کافی و کسب رضایت آگاهانه کتبی از جانب بیمار یا بستگان درجه یک (در بیماران با سطح هوشیاری پایین یا زوال عقل)، بیماران با استفاده از روش تصادفی سازی بلوک ها در دو گروه (گروه مداخله و گروه کنترل) تقسیم می شوند.

عنوان	متن
	**گروه های مداخله**<br><br>گروه A شامل بیماران سرپایی هستند که در درمان آنها علاوه بر رژیم استاندارد تعیین شده از سوی وزارت بهداشت، درمان و آموزش پزشکی ایران از داروی ایورمکتین استفاده می شود.<br><br>گروه B شامل بیماران سرپایی هستند که تنها رژیم دارویی استاندارد شامل هیدروکسی کلروکین (۴۰۰ میلی گرم دو بار در روز برای روز اول و ۲۰۰ میلی گرم دوبار در روز برای شش روز بعد) را دریافت خواهند کرد.<br><br>گروه C شامل بیماران بستری هستند که در درمان آنها علاوه بر رژیم استاندارد تعیین شده از سوی وزارت بهداشت، درمان و آموزش پزشکی ایران از داروی ایورمکتین استفاده می شود.<br><br>گروه D شامل بیمارانی بستری هستند که تنها رژیم دارویی استاندارد شامل لوپیناویر/ریتوناویر (۴۰۰ میلی گرم دو بار در روز برای روز اول و ۲۰۰ میلی گرم دوبار در روز برای شش روز بعد) را دریافت خواهند کرد.<br><br>ایورمکتین به صورت قرص های ۳ میلی گرمی (قرص استرومکتول ۳ میلی گرم، کمپانی MSD، فرانسه) در دسترس است که به صورت تک دوز (با دوز 2/0 mg/kg) تجویز می شود. در تمامی گروه ها درمان های علامتی و حمایتی مورد نیاز به صورت یکسان انجام خواهد شد.<br><br>**بررسی پیامد ها**<br><br>پیامد های اولیه این مطالعه شامل مدت زمان بستری در بیمارستان، میزان نیاز به بستری در بخش مراقب های ویژه و میزان نیاز به ونتیلاسیون برای بیماران بستری و بهبود علائم بالینی و میزان نیاز به بستری در بیمارستان برای بیماران سرپایی در طی دوره مداخله (هفت روز پس از شروع مطالعه) نظر گرفته می شود. بهبود علائم بالینی به عنوان بهبود مداوم (بیشتر از ۷۲ ساعت) درجه حرارت بدن، فرکانس تنفس و میزان اشباع اکسیژن خون پس از شروع درمان می باشد که با معیارهای کمی که در ادامه آمده است تعریف می شود: دمای دهانی ≤ ۳۶/۶ درجه سانتیگراد؛ فرکانس تنفسی ≤۲۴ بار در دقیقه و اشباع اکسیژن ≥۹۸ درصد بدون تنفس مکانیکی. علاوه بر این، نیاز به اکسیژن درمانی و تهویه با فشار مثبت غیر تهاجمی در فالوآپ های روزانه به همراه علائم بالینی دیگر علائم بالینی دیگر همچون سرفه، درد عضلانی، سردرد، تنگی نفس، ضعف و بی حالی، کاهش حس بویایی و چشایی، اسهال، اسپاسم شکمی، تهوع و استفراغ به صورت کیفی ثبت می شوند. اندازه گیری مکرر حداقل برای دو بار در هر فالوآپ انجام می شود.<br><br>پیامد های ثانویه شامل میزان بهبود پارامتر های بیوشیمیایی بیماران می باشند. انجام آزمایش های CBC[۳]، شمارش تفریقی سلول های سفید، میزان فریتین، CRP[۴]، LDH[۵]، ESR[۶]، کراتینین و نیتروژن اوره سرم در ابتدای مطالعه و انتهای مطالعه (روز ۷ مطالعه و یا زمان ترخیص) بررسی خواهد شد. همچنین عوارض جانبی ناشی از دارو های مورد مطالعه (به ویژه اسهال، اسپاسم گوارشی، تورم غدد لنفاوی، ادم دست و پا، خارش، ضعف و بی حالی شدید، تهوع و استفراغ، ادرار قرمز-قهوه ای، سردرد و سرگیجه و درد های عضلانی)،

عنوان	متن
	فراوانی عوارض جانبی احتمالی ناشی از مداخله و فراوانی انصراف از مطالعه به علت عوارض جانبی در گروه های مورد مطالعه به صورت روزانه در طی دوره مداخله (هفت روز پس از شروع مطالعه) ثبت می شوند.<br><br>[۱] Open-label randomized clinical trial<br><br>[۲] Polymerase Chain Reaction<br><br>[۳] Complete blood count<br><br>[۴] C-reactive protein<br><br>[۵] Lactate dehydrogenase<br><br>[۶] Erythrocyte sedimentation rate
جامعه مورد مطالعه	بیماران مبتلا به بیماری COVID-19
روش نمونه گیری و محاسبه اندازه نمونه	**روش محاسبه حجم نمونه**<br><br>**بیماران سرپایی**<br><br>با استفاده از مطالعات مشابه انجام شده در گروه های چند دارویی میزان بهبودی بالینی مورد انتظار در روز پنجم از گروه مورد مطالعه ۸۳ درصد، بهبودی بالینی گروه کنترل ۳۵ درصد، $\alpha = 0.05$، $\beta = 0.10$، قدرت = ۰/۹۰ در نظر گرفته شد. با توجه به توزیع ۱ :۱ بین گروه های مورد مطالعه، حجم نمونه آماری ۲۴ شرکت کننده در هر گروه محاسبه گردید. حجم نمونه با توجه به عواملی مانند ریختن نمونه ها حدود ۲۰ درصد بیشتر در نظر گرفته می شود. بنابراین این کارآزمایی شامل ۶۰ نفر (۳۰ نفر در هر گروه) می باشد.

متن	عنوان

$$N_1 = \left\{ z_{1-\alpha/2} * \sqrt{\bar{p} * \bar{q} * (1 + \frac{1}{k})} + z_{1-\beta} * \sqrt{p_1 * q_1 + (\frac{p_2 * q_2}{k})} \right\}^2 / \Delta^2$$

$$q_1 = 1 - p_1$$

$$q_2 = 1 - p_2$$

$$\bar{p} = \frac{p_1 + kp_2}{1 + K}$$

$$\bar{q} = 1 - \bar{p}$$

```
Estimated sample size for two-sample comparison of proportions

Test Ho: p1 = p2, where p1 is the proportion in population 1
                    and p2 is the proportion in population 2
Assumptions:

         alpha =   0.0500  (two-sided)
         power =   0.9000
            p1 =   0.8300
            p2 =   0.3500
         n2/n1 =   1.00

Estimated required sample sizes:

            n1 =       24
            n2 =       24
```

بیماران بستری

با استفاده از مطالعات مشابه انجام شده در گروه های چند دارویی میزان بهبودی بالینی مورد انتظار در روز پنجم از گروه مورد مطالعه ۸۳ درصد، بهبودی بالینی گروه کنترل ۳۵ درصد، $\alpha = 0.05$، $\beta = 0.10$، قدرت = ۰/۹۰ در نظر گرفته شد. با توجه به توزیع ۱ :۱ بین گروه های مورد مطالعه، حجم نمونه آماری ۲۴ شرکت کننده در هر گروه محاسبه گردید. حجم نمونه با توجه به عواملی مانند ریختن نمونه ها حدود ۲۰ درصد بیشتر در نظر گرفته می شود. بنابراین این کارآزمایی شامل ۶۰ نفر (۳۰ نفر در هر گروه) می باشد.

متن	عنوان
(see below)	

$$N_1 = \left\{ z_{1-\alpha/2} * \sqrt{\bar{p} * \bar{q} * (1 + \frac{1}{k})} + z_{1-\beta} * \sqrt{p_1 * q_1 + (\frac{p_2 * q_2}{k})} \right\}^2 / \Delta^2$$

$$q_1 = 1 - p_1$$

$$q_2 = 1 - p_2$$

$$\bar{p} = \frac{p_1 + kp_2}{1 + K}$$

$$\bar{q} = 1 - \bar{p}$$

```
Estimated sample size for two-sample comparison of proportions

Test Ho: p1 = p2, where p1 is the proportion in population 1
                    and p2 is the proportion in population 2
Assumptions:

         alpha =   0.0500  (two-sided)
         power =   0.9000
            p1 =   0.8300
            p2 =   0.3500
         n2/n1 =   1.00

Estimated required sample sizes:

            n1 =       24
            n2 =       24
```

روش تصادفی سازی بلوک

در این روش تعداد افراد در هر یک از گروه های مطالعه در طول درمان با یکدیگر برابر است. با توجه به تعداد ۶۰ نفر افراد شرکت کننده (۳۰ نفر در هر گروه) و مدت زمان تقریبی ۱۰ هفته برای تکمیل ورود افراد به مطالعه، از ۱۰ بلوک ۶ تایی (در صورت وجود بیمار به تعداد کافی از ۵ بلوک ۱۲ تایی) استفاده خواهد شد. روش کار در این نوع از تصادفی سازی شبیه به روش تصادفی سازی ساده است، تنها تعداد افراد در طول دوره درمان در دو گروه درمان یکسان می باشد. تنها ایراد این روش مشخص شدن آخرین گروه در هر بلوک می باشد.

متن	عنوان
برای تحلیل آماری از نرم افزار SPSS ورژن ۱۸/۰ استفاده می شود. برای مقایسه شاخص های اصلی اثر بخشی (کاشه بار ویروسی و میزان بهبود علائم بالینی) به عنوان پیامد اولیه و پیامد های ثانویه بین گروه های مورد مطالعه، از آزمون t برای متغیرهای کمی پیوسته و یا آزمون Wilcoxon (در صورت عدم استفاده از آزمون t) برای متغییر های کمی گسسته رتبه ای استفاده می شود. توصیف آماری متغیرهای کیفی به صورت فراوانی یا درصد مشاهده خواهد بود و برای مقایسه بین گروه ها از آزمون های Chi-square یا Fisher's exact استفاده خواهد شد. برای کلیه آزمون های آماری، P <۰،۰۵ (دو طرفه) از نظر آماری معنی دار در نظر گرفته می شود	ابزار و روشهای تجزیه و تحلیل داده ها
**ملاحظات اخلاقی**<br><br>جهت شرکت در این مطالعه از تمامی بیماران رضایت آگاهانه اخذ خواهد شد. اطلاعات مربوطه بصورت محرمانه حفظ خواهد شد. هیچ یک از مشخصات فردی افراد شرکت کننده از جمله اسم و فامیل آنها وارد کامپیوتر نخواهد شد و به تمامی افراد کد پروژه داده شده و آنالیز بر اساس آن انجام خواهد شد. اطلاعات اولیه در فایل های قفل دار و نزد مجری اصلی تا اتمام پروژه و انتشار مقالات باقی خواهد ماند. در هر مرحله از طرح بیماران می توانند بنا به تمایل شخصی از طرح خارج شوند.<br><br>**محدودیت‌های اجرایی طرح و روش رفع آن ها**<br><br>عدم همکاری بیماران که امید است با توضیحات کافی برای آن ها این مشکل رفع شود و قبل از شروع به بیماران در مورد عوارض جانبی بیماری و دارو توضیح داده می شود. از آنها قبل از شروع درمان رضایت نامه کتبی دریافت می شود. همچنین این پژوهش از نظر اخلاق پزشکی مورد تایید است. یکی از محدودیت های مهم دیگر در این طرح کمبود منابع دارویی خصوصا راجع به داروی ایورمکتین می باشد که امید است با توجه به مکاتبات لازم با معاونت غذا و دارو دانشگاه علوم پزشکی هرمزگان و مساعدت ریاست دانشکده داروسازی این مشکل رفع گردد	محدودیتهای پژوهش، خطاهای سیستماتیک احتمالی و راههای مقابله با آنها
1. Valencia DN. Brief Review on COVID-19: The 2020 Pandemic Caused by SARS-CoV-2. Cureus. 2020;12(3):e7386.<br><br>2. Dzieciatkowski T, Szarpak L, Filipiak KJ, Jaguszewski M, Ladny JR, Smereka J. COVID-19 challenge for modern medicine. Cardiology journal. 2020;27(2):175-83.<br><br>3. Wu YC, Chen CS, Chan YJ. The outbreak of COVID-19: An overview. Journal of the Chinese Medical Association : JCMA. 2020;83(3):217-20.<br><br>4. Caly L, Druce JD, Catton MG, Jans DA, Wagstaff KM. The FDA-approved drug ivermectin inhibits the replication of SARS-CoV-2 in vitro. Antiviral research. 2020;178:104787.	منابع

متن	عنوان
5. Choudhary R, Sharma AK. Potential use of hydroxychloroquine, ivermectin and azithromycin drugs in fighting COVID-19: trends, scope and relevance. New microbes and new infections. 2020;35:100684.<br><br>6. Rizzo E. Ivermectin, antiviral properties and COVID-19: a possible new mechanism of action. Naunyn-Schmiedeberg's archives of pharmacology. 2020;393(7):1153-6.<br><br>7. Velavan TP, Meyer CG. The COVID-19 epidemic. Trop Med Int Health. 2020;25(3):278-80.<br><br>8. Hui DS, Azhar EI, Madani TA, Ntoumi F, Kock R, Dar O, et al. The continuing 2019-nCoV epidemic threat of novel coronaviruses to global health—The latest 2019 novel coronavirus outbreak in Wuhan, China. International Journal of Infectious Diseases. 2020;91:264.<br><br>9. Wu Z, McGoogan JM. Characteristics of and important lessons from the coronavirus disease 2019 (COVID-19) outbreak in China: summary of a report of 72 314 cases from the Chinese Center for Disease Control and Prevention. Jama. 2020.<br><br>10. Colson P, Rolain J-M, Lagier J-C, Brouqui P, Raoult D. Chloroquine and hydroxychloroquine as available weapons to fight COVID-19. Int J Antimicrob Agents. 2020;105932(10.1016).<br><br>11. Colson P, Rolain J-M, Raoult D. Chloroquine for the 2019 novel coronavirus. Int J Antimicrob Agents. 2020.<br><br>12. Cao B, Wang Y, Wen D, Liu W, Wang J, Fan G, et al. A Trial of Lopinavir-Ritonavir in Adults Hospitalized with Severe Covid-19. The New England journal of medicine. 2020;382(19):1787-99.	

جدول متغیرها

متغیر	نحوه اندازه گیری	واحد اندازه گیری	نوع متغیر	نقش متغیر	تعریف علمی/عملی	توضیحات
بیماران مبتلا به بیماری COVID-19	پرسشنامه		کیفی-اسمی	مستقل		
درجه حرارت بدن	ترمومتر	درجه حرارت بدن	کمی/پیوسته	وابسته	دمای (دهانی) بیشتر از ۶/۳۶ سانتیگراد به عنوان تب در نظر گرفته می شود.	
دمای (دهانی) بیشتر از ۶/۳۶ سانتیگراد به عنوان تب در نظر گرفته می شود.	شمارش	تعداد در دقیقه	کمی/گسسته	وابسته	دمای (دهانی) بیشتر از ۶/۳۶ سانتیگراد به عنوان تب در نظر گرفته می شود.	
اکسیژن خون	پالس اکسی متر	درصد	کمی/گسسته	وابسته	اشباع اکسیژن بیشتر از ۹۸ درصد بدون تنفس مکانیکی به عنوان اشباع نرمال در نظر گرفته می شود.	
سرفه	پرسشنامه		کیفی-اسمی	وابسته		
سرفه	پرسشنامه		کیفی-اسمی	وابسته		
کاهش حس بویایی و چشایی	پرسشنامه		کیفی-اسمی	وابسته		
ضعف و بی حالی	پرسشنامه		کیفی-اسمی	وابسته		
سن	بر اساس تاریخ تولد شخص یا گفته ی خود شخص	سال	کمی/گسسته	زمینه ای		
جنسیت	پرسشنامه		کیفی-اسمی	زمینه ای		

متغیر	نحوه اندازه گیری	واحد اندازه گیری	نوع متغیر	نقش متغیر	تعریف علمی/عملی	توضیحات
شغل	پرسشنامه		کیفی-اسمی	زمینه ای		
CBC	آزمایشگاهی	mg/L	کمی/پیوسته	وابسته	Complete blood count	
LDH	آزمایشگاهی	µmol/L	کمی/پیوسته	وابسته	Lactate dehydrogenase	
ESR	آزمایشگاهی	mm/hour	کمی/پیوسته	وابسته	Erythrocyte sedimentation rate	
SeCr	آزمایشگاهی	g/mol	کمی/پیوسته	وابسته	Serum Creatinine	
BUN	آزمایشگاهی	mg/dl	کمی/پیوسته	وابسته	Blood urea nitrogen	
